# Multiple Myeloma and Secondary Immunodeficiency: A Retrospective Database Analysis Assessing Burden of Infection and Treatment Patterns

**DOI:** 10.1155/ah/5340241

**Published:** 2025-12-25

**Authors:** Csaba Siffel, Matthew S. Davids, Colin Anderson-Smits, Marta Kamieniak, Kaili Ren, Shirin Ardeshir-Rouhani-Fard, Drishti Shah, Joshua Richter

**Affiliations:** ^1^ Global Evidence and Outcomes, Data and Quantitative Sciences Institute, Takeda Development Center Americas, Inc., Cambridge, Massachusetts, USA; ^2^ Department of Interdisciplinary Health Science, College of Allied Health Sciences, Augusta University, Augusta, Georgia, USA, augusta.edu; ^3^ Department of Medical Oncology, Dana-Farber Cancer Institute, Boston, Massachusetts, USA, dana-farber.org; ^4^ Global Medical Affairs, Takeda Development Center Americas, Inc., Cambridge, Massachusetts, USA; ^5^ Statistics and Quantitative Sciences, Data and Quantitative Sciences Institute, Takeda Development Center Americas, Inc., Cambridge, Massachusetts, USA; ^6^ Department of Hematology and Medical Oncology, Tisch Cancer Institute, Icahn School of Medicine at Mount Sinai, New York, New York, USA, mountsinai.org

**Keywords:** database, immunoglobulin G replacement therapy, infections multiple myeloma, secondary immunodeficiency

## Abstract

Patients with multiple myeloma (MM) and secondary immunodeficiency (SID) are at risk of infection‐related morbidity and mortality owing to the disease process and treatment toxicity. This retrospective cohort study used anonymized data from the Optum‐Humedica database (October 2015–March 2020) to assess the burden of infection in patients with MM and SID versus patients without SID. Patients aged ≥ 18 years with confirmed MM were assigned to SID and no‐SID cohorts using an algorithm considering serum IgG levels < 5.0 g/L, hypogammaglobulinemia diagnosis codes, and ≥ 1 major infection. Patients with SID were stratified into those treated or not treated with immunoglobulin replacement therapy (IgRT and no‐IgRT cohorts). At 12 months follow‐up, a greater proportion of the SID cohort than the no‐SID cohort experienced infections (58.9% vs. 31.3%; *p* < 0.001), severe infections (29.8% vs. 10.6%, *p* < 0.001), and infection‐related hospitalizations (26.9% vs. 9.1%; *p* < 0.001). Bacterial infections were the most common infection type (SID cohort, 49.2%; no‐SID cohort, 26.1%; IgRT cohort, 70.4%; no‐IgRT cohort, 45.1%). Use of anti‐infectives and healthcare resource utilization was higher in the SID cohort than in the no‐SID cohort. Median overall survival was shorter in the SID versus no‐SID cohort (12.6 vs. 14.7 months; *p* < 0.001) and was similar in the IgRT versus no‐IgRT cohort (8.5 vs. 12.5 months; *p* = 0.446). Among patients with MM and SID, the higher infection burden in patients treated with IgRT than no‐IgRT suggests the IgRT cohort was a more vulnerable population. A better understanding of SID burden may improve outcomes for patients with MM.

## 1. Introduction

Owing to the course of the underlying disease and the immunosuppressive effects of anticancer therapies, patients with mature B‐cell malignancies, such as multiple myeloma (MM), are at risk of developing secondary immunodeficiency (SID) [1–4]. MM is the most common hematological malignancy after lymphoma [4, 5] and is the cause of 20% of all deaths related to blood disorders [4]. In the United States, the incidence of MM was 7.2 per 100,000 individuals per year over the period 2017–2021, and 5‐year relative survival was 61.1% based on 2014–2020 data [6]. Characteristics of MM include dysfunctional proliferation of plasma cells in the bone marrow, production of monoclonal antibody (M‐protein) [7], and disrupted production of normal immunoglobulins [8], resulting in immune dysregulation [9, 10].

SID manifests as severe, recurrent, or persistent infections, and patients with MM are at high risk of both bacterial and viral infections, especially in the first year after diagnosis [11]. There are several factors that increase the risk of infection in MM, including the presence of hypogammaglobulinemia; advancing age; comorbidities (e.g., heart and lung disease, kidney failure); steroid‐induced immunosuppression; mucositis and bone marrow failure caused by cytotoxic treatment; and the cumulative effects of the principal disease and treatment that can decrease antibody response to infections and vaccines and lead to T‐cell, dendritic cell, and natural killer cell dysfunction (related to anti‐CD38 antibody treatment) [3, 4, 12]. In addition, new therapies like chimeric antigen receptor T‐cell (CAR‐T) and bispecific antibody treatments, especially those targeting the B‐cell maturation antigen (BCMA) receptor, can substantially increase the risk of infection in patients with MM [13]. Both treatments have been attributed to several life‐threatening complications, such as increased risk of serious infections, hypogammaglobulinemia, cytokine release syndrome, immune effector cell‐associated neurotoxicity syndrome, neutropenia, lymphopenia, and T‐cell exhaustion [13–15]. Treatment‐emergent infections are commonly observed, and therefore, it is crucial to consider the risk and type of infection in individual patients when selecting an appropriate cancer treatment [12].

Patients with SID often present with hypogammaglobulinemia or more subtle immunoglobulin G (IgG) subclass deficiencies or functional antibody deficiencies in the presence of normal total IgG levels [16]. Hypogammaglobulinemia may occur in up to 90% of patients with MM, depending on the disease subtype [17]. Delay in diagnosis of antibody deficiencies has been associated with greater infection‐related morbidity [18, 19]. For patients with SID, it is crucial to promptly diagnose and treat in order to minimize disease progression and to avoid infection‐related morbidity and mortality [2, 16].

In clinical practice, patients with mature B‐cell malignancies and SID are often managed using antibiotics and/or immunoglobulin replacement therapy (IgRT) for infection prevention [17, 20]. IgRT may be warranted for patients with low serum IgG levels (i.e., < 4.0 g/L) who continue to experience severe, persistent, or recurrent infections, despite use of antibiotics, and for those in whom functional antibody deficiency has been identified via inadequate vaccine responses [2, 17, 21–23]. Although the use of IgRT is well established in primary immunodeficiency diseases [20], there is a paucity of evidence on the efficacy of IgRT in SID from randomized controlled trials [24]; most studies were completed in the 1990s [25–29], with the most recent randomized controlled trial of IgRT specifically in patients with MM being completed in 2018 [30].

Despite the substantial morbidity and mortality associated with MM, there is currently limited guidance to help clinicians to identify and treat patients at the highest risk of developing SID and subsequent infections [1, 31]. Prophylactic IgRT is not routinely recommended in MM [32, 33], but may be considered in patients with severe recurrent bacterial infections and hypogammaglobulinemia [17, 33]. However, prophylactic IgRT is recommended for patients with MM with a serum IgG < 4 g/L, treated with CAR‐T cell therapy or bispecific antibody therapy [13, 34, 35]. An international online survey of physicians responsible for the diagnosis of SID and the prescription of IgRT revealed regional and specialty‐related differences in clinical practice when managing and treating MM Reference [20]. This reflected a lack of consensus between specialists as to the optimal management approach for patients with MM and SID [20, 36].

Given the general paucity of evidence on this topic, the aim of the current study was to collect data that comprehensively describe the burden of infection experienced by patients with MM and SID compared with those without SID. These data included the demographics, clinical characteristics, numbers, types, and severity of infections, healthcare resource utilization (HCRU), treatment patterns, and overall survival. Furthermore, to assess the impact of IgRT use, these outcomes were also compared between patients with SID who were treated or not treated with IgRT.

## 2. Methods

### 2.1. Study Design and Population

This retrospective, observational, cohort study used anonymized data from the Optum‐Humedica electronic record database in the United States from October 1, 2015, to March 10, 2020 (Figure S1). The analysis described herein was part of a broader analysis to assess outcomes in different B‐cell malignancies (chronic lymphocytic leukemia/small lymphocytic lymphoma, MM, and non‐Hodgkin’s lymphoma), with or without SID, the results of which have been reported elsewhere [37]. An analysis, derived from this broader analysis of patients with chronic lymphocytic leukemia/small lymphocytic lymphoma with or without SID, treated with or without IgRT, has also been published [38]. In the current analysis, diagnosis of MM was defined as having at least two International Classification of Diseases, 10th Revision, Clinical Modification (ICD‐10‐CM) diagnosis codes for MM (C90.0: not having achieved remission, C90.00; in remission, C90.01; in relapse, C90.02), occurring at least 30 days apart in the 6‐month pre‐index period (PIP). The index date for the SID cohort was the date of the first SID record, defined as the earliest occurrence of an ICD‐10‐CM code for hypogammaglobulinemia or a record of serum total IgG levels < 5.0 g/L [39]. The index date for the no‐SID cohort was a randomly assigned date to replicate the distribution of index dates in the SID cohort. Patients with and without SID were identified from April 1, 2016, to March 10, 2019 (selection window). Full details of the SID operational definition have been described previously [37]. Briefly, patients in the SID cohort were defined as having either serum IgG levels < 5.0 g/L, specific antibody failure, ICD‐10‐CM codes for non‐familial hypogammaglobulinemia (D80.1), selective IgG deficiency (D80.3) or antibody deficiency with near‐normal immunoglobulins (D80.6), and the presence of at least one major infection during the selection window. Major infections were defined as bacteremia or sepsis, bacterial meningitis, osteomyelitis/septic arthritis, bacterial pneumonia, visceral abscess, or opportunistic infections. These opportunistic infections included listeriosis, fungal infections, and viral infections (including cytomegalovirus, hepatitis B virus, and hepatitis C virus). Patients in the no‐SID cohort were defined as those who did not meet the inclusion criteria for the SID cohort (based on a predefined algorithm) but who met the criteria for MM diagnosis during the PIP. Patients with evidence of SID during the PIP were excluded to ensure inclusion of only patients with newly diagnosed SID. Patients with primary immunodeficiency disease during the PIP were also excluded.

Eligible patients were aged 18 years and older at the index date, with MM clinical activity (defined as at least one medical or pharmacy claim of any type, as a proxy for continuous enrollment) for at least 6 months before and for at least 3 months after the index date (ending at the first occurrence of last alive date or end of study date). Full details of inclusion and exclusion criteria have been described previously [37].

Patients with SID were further stratified according to whether IgRT was received. Patients receiving IgRT (IgRT cohort) were re‐indexed to the date upon which IgRT was first received after the SID index date. All patients who were not assigned an IgRT index date during the selection window were included in the no‐IgRT cohort. As patients in this group did not have an IgRT‐related index date, a random pseudo‐index date was assigned, based on the distribution of the IgRT index date of patients in the SID with the IgRT subgroup. For these cohorts, eligible patients showed clinical activity for at least 3 months after the IgRT index/pseudo‐index date (minimum 3‐month and variable 12‐month follow‐up period) and for 6 months before the IgRT index/pseudo‐index date, that is, during the PIP. For the IgRT cohort, patients were required to have had repeated IgRT exposure (defined as receipt of IgRT on two separate dates between the IgRT index date and the end of the study period). To ensure only IgRT‐naive patients were included, patients with any IgRT use before the IgRT index/pseudo‐index date in the 6‐month PIP were excluded.

### 2.2. Outcomes

The following outcomes were assessed using data from patients with ≥ 12 months of follow‐up for both the SID and no‐SID cohorts and the IgRT and no‐IgRT cohorts: baseline demographics and clinical characteristics (both reported on the index date or as close to the index date as possible); number, type, and severity of infections and bacterial infections; HCRU (infection‐associated hospitalizations, inpatient admissions, outpatient services utilization, and pharmacy utilization); and treatment patterns (anti‐infectives, anticancer treatments, and supportive care agents). For all cohorts, patients were followed for a variable follow‐up period (defined as time starting on and after the index date, ending at the first occurrence of either the end of the study period [March 10, 2020] or death, with a minimum of 3 months of continuous eligibility required).

Severe infections were defined as no response to oral antibiotics (defined as a switch in antibiotics or the addition of another antibiotic to the patient’s regimen); presence of at least one claim for an intravenous antibiotic or presence of at least one claim for at least two oral antibiotics; hospitalization with any ICD‐10‐CM code for infection; infection with an unusual pathogen (*Clostridium difficile*, *Neisseria* sp., *Giardia* sp.); or unusual complications (e.g., mastoiditis, pleural effusion, abscess). Severe bacterial infections (SBIs) were defined using ICD‐10‐CM codes for bacteremia or sepsis, bacterial meningitis, osteomyelitis/septic arthritis, bacterial pneumonia, and visceral abscess. Full details of the ICD‐10‐CM diagnosis codes for SBIs can be found in Table S1. Overall survival was assessed in patients with at least 3 months of follow‐up. In addition to the analysis of the burden of infection, the following clinical and treatment‐related outcomes were also assessed for the IgRT and no‐IgRT cohorts, where available, among those patients with a minimum of 12‐month follow‐up data: number and severity of infections, antimicrobial use, length of hospitalizations, and serum IgG levels.

### 2.3. Statistical Analysis

Descriptive statistics were used for all relevant study outcomes. Bivariate comparisons between the SID and no‐SID cohorts were conducted using parametric independent sample *t*‐tests (mean) and nonparametric Wilcoxon rank‐sum tests (median) for continuous variables and chi‐square tests or Fisher’s exact tests for categorical variables. A *p-*value of < 0.05 was considered statistically significant. Time‐to‐event measures (overall survival/time to death) were described using Kaplan–Meier analysis.

Similar statistical analyses were performed for the IgRT and no‐IgRT cohorts. To adjust for selection bias and potential confounding factors, patients in the SID IgRT cohort were matched on a 1:1 basis via propensity score matching to patients in the SID no‐IgRT cohort. Matching was first performed for all hematological malignancies that were included in the initial broader analysis. For the purposes of this analysis, *post hoc* matching was subsequently performed for MM alone with variables determined after review of descriptive, unmatched results. The variables included were Charlson Comorbidity Index (CCI) score, chronic obstructive pulmonary disease, cytopenia, pre‐index antibiotics, age, duration between SID index date and treatment index date, duration of cancer, any infection, and secondary/other malignancies. The propensity scores were calculated by summing coefficient values for potential confounding variables. Propensity scores were generated using a multivariable logistic regression model in which the dependent variable was IgRT use (yes/no). Standardized mean differences (SMDs) were reported for the unmatched and matched cohorts as a measure of balance. SMD was calculated as the difference in means or proportions of a variable divided by the pooled standard deviation (SD). An SMD of ≥ 0.10 between groups was considered meaningful and would indicate imbalance. For matched cohorts, bivariate comparisons were conducted using parametric paired *t*‐tests (mean) and nonparametric Wilcoxon signed‐rank tests (median) for continuous variables and the McNemar’s test for categorical variables.

## 3. Results

### 3.1. Patient Disposition

Overall, 4592 patients had MM with ≥ 12‐month follow‐up (Figure S2); 890 and 3702 patients were included in the SID and no‐SID cohorts, respectively. Before matching, 71 and 669 patients were included in the IgRT and no‐IgRT cohorts, respectively. Analyses of matched IgRT and no‐IgRT cohorts were performed using data from 71 patients from each cohort.

### 3.2. Baseline Demographics and Clinical Characteristics

Baseline demographics of the SID and no‐SID cohorts are summarized in Table S2. Compared with the no‐SID cohort, the SID cohort had higher mean (SD) CCI scores (4.2 [2.26] vs. 3.5 [1.93]; *p* < 0.001) and a shorter mean (SD) duration of MM (13.0 [10.26] vs. 15.3 [10.07] months; *p* < 0.001). Compared with the no‐SID cohort, a significantly higher proportion of patients in the SID cohort had experienced an infection (41.3% vs. 22.6%; *p* < 0.001), and the same trend was observed for SBIs (20.0% vs. 9.0%; *p* < 0.001). Patients in the SID cohort had more exposure to anti‐infectives (84.8% vs. 47.3%; *p* < 0.001) and to immunosuppressants (0.7% vs. 0.3%; *p* = 0.119) than patients in the no‐SID cohort.

Baseline demographics and clinical characteristics of the unmatched and matched IgRT and no‐IgRT cohorts are summarized in Table S3. Before matching, numerical imbalances between the cohorts were noted with regard to distribution in the categories for race, ethnicity, CCI score, duration of MM, infections and SBIs, exposure to anti‐infectives, and exposure to immunosuppressants (all SMDs > 0.1). After propensity score matching, the mean (SD) CCI score reported in patients in both cohorts was similar (4.8 [2.43] vs. 4.7 [2.30]; *p* = 0.859; SMD 0.03). However, after matching, a significantly greater proportion of patients in the IgRT cohort had experienced an SBI (46.5% vs. 29.6%; *p* = 0.028; SMD 0.35), although the proportions of patients exposed to anti‐infectives (97.2% vs. 95.8%; *p* = 0.564; SMD 0.08) and immunosuppressants (4.2% vs. 2.8%; *p* = 0.655; SMD 0.08) were similar between cohorts.

### 3.3. Infections

Infection‐related outcomes are presented in Figure 1. At 12‐month follow‐up, infection or severe infection was experienced by a greater proportion of patients in the SID cohort than in the no‐SID cohort (Figure 1(a)) and in the IgRT cohort than in the no‐IgRT cohort (Figure 1(b)). Furthermore, at 12‐month follow‐up, there was a significantly higher mean (SD) number of infections in the SID cohort than in the no‐SID cohort (7.3 [9.43] vs. 4.8 [7.22]; *p* < 0.001) and in the IgRT cohort than in the no‐IgRT cohort (11.9 [11.44] vs. 8.0 [10.66]; *p* = 0.140). In addition, there was a significantly higher mean (SD) number of severe infections in the SID cohort than in the no‐SID cohort (6.4 [8.63] vs. 4.8 [5.92]; *p* = 0.008); however, no statistically significant difference was observed between the matched IgRT and no‐IgRT cohorts (10.0 [10.09] vs. 5.9 [4.19]; *p* = 0.203).

Figure 1Occurrence of infection (a, b) and types of infection (c, d), and occurrence of SBIs (e) and types of SBIs (f, g) in the SID and no‐SID cohorts and in the IgRT and no‐IgRT cohorts at 12‐month follow‐up. For panel d, one patient (1.4%) in the IgRT cohort experienced parasitic infection; no patients in the no‐IgRT cohort had experienced a parasitic infection. For panel g, one patient in the IgRT cohort had experienced bacterial meningitis; none of the patients in the no‐IgRT cohort had experienced bacterial meningitis. No patients in the IgRT and no‐IgRT cohorts experienced a visceral abscess. IgRT, immunoglobulin replacement therapy; SBI, severe bacterial infection; SID, secondary immunodeficiency.

**Figure:**
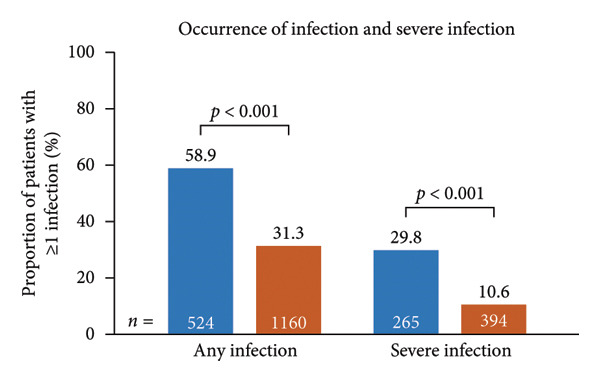
(a)

**Figure:**
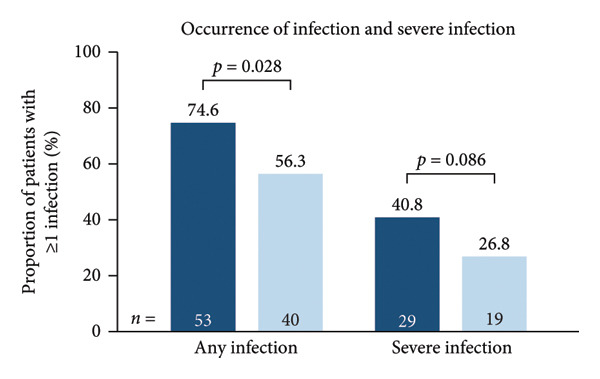
(b)

**Figure:**
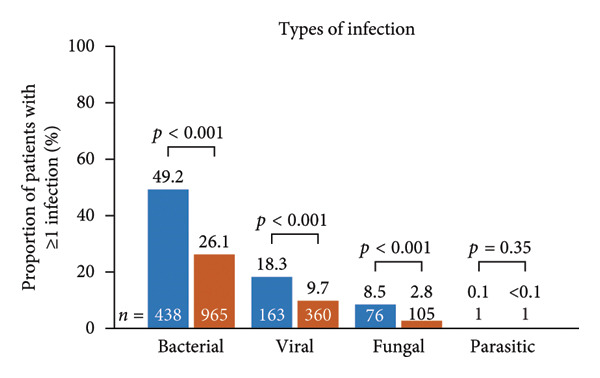
(c)

**Figure:**
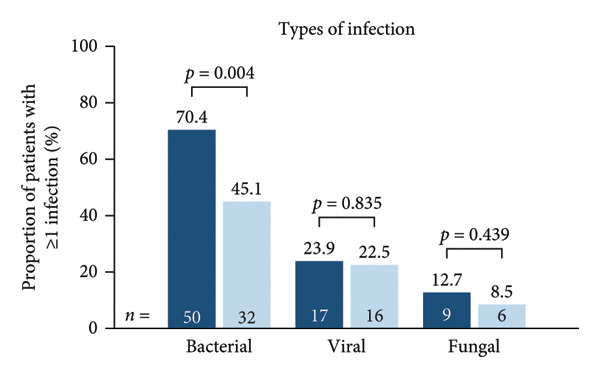
(d)

**Figure:**
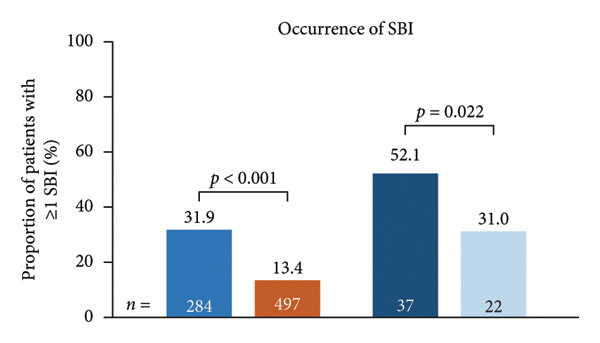
(e)

**Figure:**
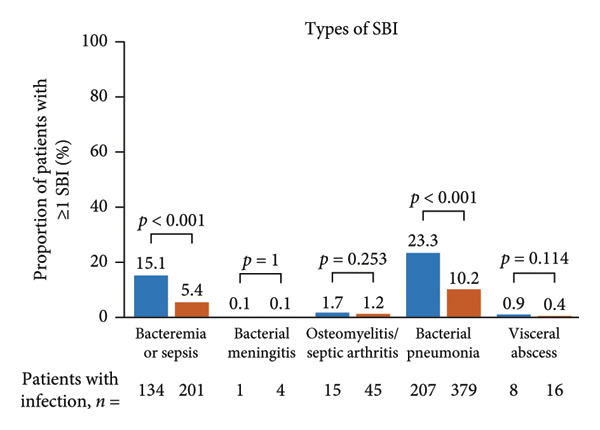
(f)

**Figure:**
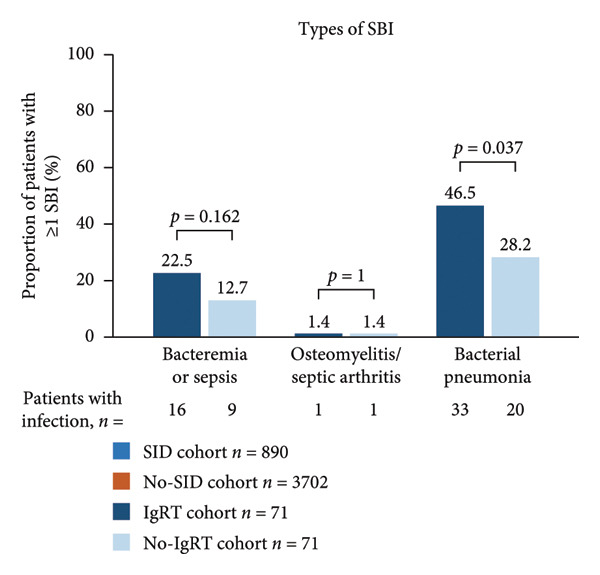
(g)

The most common type of infection in all cohorts was bacterial (Figures 1(c) and 1(d)). At 12‐month follow‐up, the proportion of patients experiencing at least one SBI was significantly greater in the SID cohort than in the no‐SID cohort and in the IgRT cohort than in the no‐IgRT cohort (Figure 1(e)). In addition, a higher mean (SD) number of SBIs was reported in the SID cohort than in the no‐SID cohort (7.1 [7.74] vs. 5.9 [8.56]; *p* = 0.042); however, no significant difference was observed between the matched IgRT and no‐IgRT cohorts (9.3 [9.53] vs. 6.6 [7.15]; *p* = 0.568). In those patients who experienced an SBI, the most frequently reported in all cohorts were bacterial pneumonia and bacteremia or sepsis (Figures 1(f) and 1(g)).

### 3.4. HCRU

HCRU data for all cohorts are summarized in Table 1. Compared with the no‐SID cohort, the SID cohort had significantly higher proportions of patients who had at least one all‐cause or infection‐associated hospitalization; hospital stays were also longer in the SID cohort. The SID cohort also had significantly greater proportions of patients who visited the emergency room or had a physician’s office, laboratory/pathology, surgical service, or radiology visit, and had a higher number of prescription fills. In contrast, there were no clear differences in HCRU between the IgRT and no‐IgRT cohorts.

**Table 1:** HCRU and treatment patterns at 12‐month follow‐up.

**HCRU**							
**Patients with ≥ 1 of the listed healthcare resources, *n* (%)** ^**a**^	**SID cohort** **(*n* = 890)**	**No-SID cohort** **(*n* = 3702)**	***p* value**	**IgRT cohort** **(*n* = 71)**		**No-IgRT cohort** **(*n* = 71)**	***p* value**

Inpatient all‐cause hospitalizations	433 (48.7)	690 (18.6)	< 0.001	35 (49.3)		29 (40.8)	0.303
Number per patient, mean (SD)	2.0 (1.62)	1.6 (1.10)	< 0.001	2.3 (2.29)		2.0 (1.75)	0.131
LOS, days, mean (SD)	11.5 (25.89)	8.6 (22.42)	0.056	17.3 (60.86)		9.5 (10.15)	0.122
Infection‐associated hospitalizations	239 (26.9)	338 (9.1)	< 0.001	27 (38.0)		17 (23.9)	0.077
Number per patient, mean (SD)	6.8 (8.90)	5.3 (6.12)	0.021	10.1 (10.29)		5.9 (4.04)	0.206
LOS, days, mean (SD)	12.9 (34.37)	8.6 (15.75)	0.071	20.2 (69.24)		8.4 (7.41)	0.419
ER visit	343 (38.5)	936 (25.3)	< 0.001	22 (31.0)		28 (39.4)	0.289
Number per patient, mean (SD)	2.4 (2.98)	1.9 (2.03)	0.007	3.1 (2.83)		2.0 (1.32)	0.449
Physician’s office visit	779 (87.5)	2996 (80.9)	< 0.001	63 (88.7)		63 (88.7)	1
Number per patient, mean (SD)	22.4 (19.52)	14.6 (14.44)	< 0.001	26.1 (21.13)		21.5 (20.05)	0.178
Laboratory/pathology visit	890 (100.0)	3517 (95.0)	< 0.001	71 (100.0)		70 (98.6)	NA
Number per patient, mean (SD)	38.8 (24.92)	18.3 (17.54)	< 0.001	42.8 (27.76)		36.9 (27.48)	0.197
Surgical service visit	41 (4.6)	80 (2.2)	< 0.001	7 (9.9)		3 (4.2)	0.206
Number per patient, mean (SD)	1.3 (0.50)	1.4 (0.72)	0.402	1.1 (0.38)		2.0 (1.00)	NA
Radiology service visit	51 (5.7)	37 (1.0)	< 0.001	3 (4.2)		8 (11.3)	0.132
Number per patient, mean (SD)	1.4 (0.66)	1.5 (1.02)	0.383	2.0 (1.73)		1.0 (0.00)	NA
Prescription fill	883 (99.2)	3279 (88.6)	< 0.001	71 (100.0)		70 (98.6)	NA
Number per patient, mean (SD)	42.6 (27.78)	22.2 (20.66)	< 0.001	47.6 (31.12)		41.9 (25.96)	0.262

**Treatment patterns**							
**Patients with any use of the listed treatments, *n* (%)** ^**a**^	**SID cohort** **(*n* = 890)**	**No-SID cohort** **(*n* = 3702)**	***p* value**		**IgRT cohort** **(*n* = 71)**	**No-IgRT cohort** **(*n* = 71)**	***p* value**

Any anti‐infective use	822 (92.4)	2163 (58.4)	< 0.001		65 (91.5)	60 (84.5)	0.225
Any antibiotic use	769 (86.4)	1769 (47.8)	< 0.001		61 (85.9)	59 (83.1)	0.67
Number of claims, mean (SD)	9.0 (9.61)	4.6 (6.44)	< 0.001		10.3 (10.05)	7.3 (7.28)	0.142
IV antibiotic use	479 (53.8)	758 (20.5)	< 0.001		37 (52.1)	31 (43.7)	0.317
Number of claims, mean (SD)	6.6 (7.57)	4.8 (6.49)	< 0.001		8.1 (7.14)	6.8 (6.28)	0.103
Any antiviral use	667 (74.9)	1205 (32.5)	< 0.001		56 (78.9)	50 (70.4)	0.303
Number of claims, mean (SD)	10.0 (12.71)	4.5 (6.23)	< 0.001		8.3 (13.54)	8.8 (10.56)	0.859
IV antiviral use	6 (0.7)	0	< 0.001		0	2 (2.8)	NA
Number of claims, mean (SD)	8.8 (10.27)	0	NR		0	18.0 (24.04)	NA
Any antifungal use	251 (28.2)	201 (5.4)	< 0.001		13 (18.3)	14 (19.7)	0.819
Number of claims, mean (SD)	10.4 (11.45)	5.3 (6.34)	< 0.001		14.2 (16.96)	7.6 (9.26)	0.713
IV antifungal use	44 (4.9)	27 (0.7)	< 0.001		4 (5.6)	4 (5.6)	1
Number of claims, mean (SD)	6.4 (5.28)	6.4 (5.32)	0.95		3.8 (2.06)	3.0 (2.45)	NR
Any chemotherapeutic agent use	712 (80.0)	1693 (45.7)	< 0.001		61 (85.9)	54 (76.1)	0.144
Number of claims, mean (SD)	21.0 (15.93)	16.1 (13.28)	< 0.001		21.3 (17.10)	23.9 (16.33)	0.379
Any use of a supportive care agent	6 (0.7)	4 (0.1)	0.005		1 (1.4)	0	NA
Number of claims, mean (SD)	5.2 (5.35)	1.8 (0.50)	0.179		12.0 (NA)	0	NA

*Note:* The mean (SD) number of each healthcare resource type per patient was calculated among patients with any use of that resource type. The mean (SD) number of claims for each treatment type was calculated among patients with any use of that treatment type.

Abbreviations: ER = emergency room, HCRU = healthcare resource utilization, IgRT = immunoglobulin replacement therapy, IV = intravenous, LOS = length of stay, NA = not available, NR = not reported, SD = standard deviation, SID = secondary immunodeficiency.

^a^Unless otherwise stated.

### 3.5. Treatment Patterns

Treatment patterns in all cohorts are summarized in Table 1. Compared with the no‐SID cohort, a significantly higher proportion of patients with SID used an anti‐infective; antibiotics were the most common type of anti‐infective used. In addition, a significantly higher proportion of patients with SID received a chemotherapeutic agent compared with the no‐SID cohort. For the matched IgRT cohort, the median duration of IgRT was 258.0 days, with a median of 6.0 IgRT prescriptions filled per patient. Treatment patterns were not significantly different between the IgRT and no‐IgRT cohorts (Table 1).

### 3.6. Overall Survival

Among patients with a minimum of 3 months’ follow‐up, 297 deaths (25.9%) occurred in the SID cohort compared with 921 deaths (19.4%) in the no‐SID cohort. Overall survival was shorter in the SID cohort than in the no‐SID cohort (*p* < 0.001), with a median time to death of 12.6 and 14.7 months, respectively (Figure 2(a)). This trend was observed at all time points assessed (probability of survival [95% confidence intervals] at 6 months: 95.1% [93.6, 96.2] vs. 97.2% [96.7, 97.6]; 12 months: 87.6% [85.5, 89.4] vs. 91.6% [90.7, 92.4]; 24 months: 74.0% [70.9, 76.8] vs. 82.2% [80.9, 83.4]).

Figure 2Kaplan–Meier curves for overall survival in the SID and no‐SID cohorts (a) and in the IgRT and no‐IgRT cohorts of patients with SID (b). The number of patients at risk and 95% Hall–Wellner bands are shown. IgRT, immunoglobulin replacement therapy; pts, patients; SID, secondary immunodeficiency.

**Figure:**
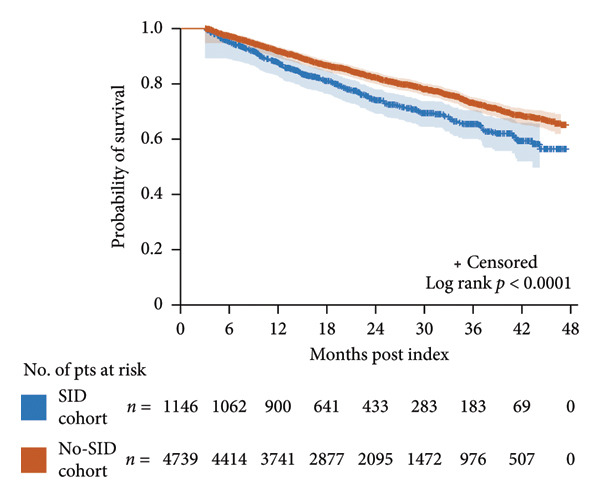
(a)

**Figure:**
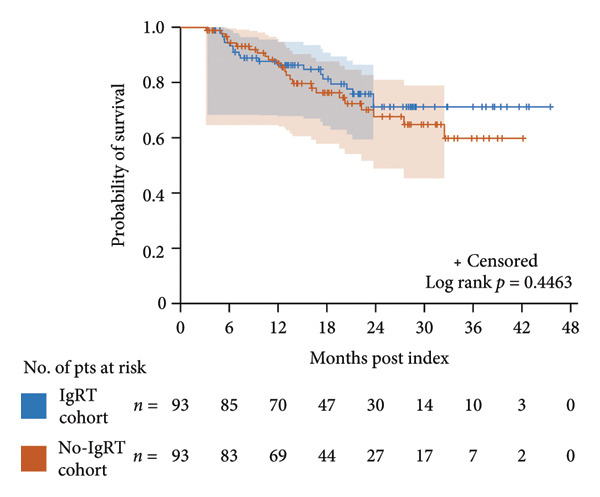
(b)

In the IgRT and no‐IgRT cohorts, there were 20 deaths (21.5%) and 24 deaths (25.8%), respectively, with a median time to death of 8.5 and 12.5 months. The probability of survival was lower in the no‐IgRT cohort than in the IgRT cohort after 12 months; however, 95% confidence intervals overlapped at 6 and 12 months, and a comparable overall survival was observed between patients with SID in the IgRT and no‐IgRT cohorts (*p* = 0.446) (Figure 2(b)).

### 3.7. Clinical and Treatment Response Outcomes by IgRT Cohort

Clinical and treatment response outcomes in the matched IgRT and no‐IgRT cohorts of patients with SID are provided in Table 2. The mean rate of infections during the 12‐month follow‐up period was almost twice as high in the IgRT cohort than in the no‐IgRT cohort (8.9 vs. 4.5, respectively). Between‐cohort differences were smaller for the incidences of hospitalizations and severe infections, although the incidences of all clinical and treatment response outcomes were higher in the IgRT cohort than in the no‐IgRT cohort. Among patients with available data, fewer patients in the IgRT cohort than in the no‐IgRT cohort maintained an IgG level of < 5.0 g/L after index and throughout the 12‐month follow‐up period: 80.3% of IgRT‐treated patients had an increase in IgG serum levels to ≥ 5.0 g/L over the follow‐up period compared with 67.6% of the no‐IgRT cohort.

**Table 2:** Clinical and treatment response outcomes for IgRT‐treated and no‐IgRT matched cohorts of patients with SID at 12‐month follow‐up.

	IgRT (*n* = 71)	No‐IgRT (*n* = 71)	Difference^a^	IRR^b^
Any infections				
Number per patient, mean (SD)	8.9 (11.16)	4.5 (8.89)		
Incidence^c^	8.90	4.48	−4.42	1.99
Severe infections				
Number per patient, mean (SD)	4.1 (8.09)	1.6 (3.38)		
Incidence	4.10	1.58	−2.52	2.59
Antimicrobial use				
Number per patient, mean (SD)	18.0 (28.93)	13.7 (18.90)		
Incidence	18.04	13.72	−4.32	1.31
Hospitalizations				
Number per patient, mean (SD)	1.1 (1.97)	0.8 (1.49)		
Incidence	1.14	0.82	−0.32	1.39
Length of hospitalizations				
Mean (SD), days	13.1 (44.96)	6.2 (11.42)		
Serum IgG levels, *n* (%)				
Maintained level of < 5.0 g/L^d^	6 (8.5)	15 (21.1)		
Increased level to ≥ 5.0 g/L	57 (80.3)	48 (67.6)		
Missing data	8 (11.3)	8 (11.3)		

Abbreviations: IgG = immunoglobulin G; IgRT = immunoglobulin replacement therapy; IRR = incidence rate ratio; SD = standard deviation; SID = secondary immunodeficiency.

^a^Measured as the difference between the two cohorts: IgRT − no‐IgRT.

^b^Measured as IgRT ÷ no‐IgRT.

^c^Incidence was calculated for the 12‐month period beginning at the IgRT index date.

^d^Serum IgG level recorded at index date.

## 4. Discussion

This study explored real‐world evidence to gain a better understanding of the clinical burden of SID among patients with MM in the United States and the treatment outcomes associated with IgRT use in patients with SID. Results from this study demonstrate that patients with MM who developed SID had a higher burden of infection and HCRU than those without SID. In addition, infection burden was higher among IgRT‐treated patients with SID than among patients in the no‐IgRT cohort; however, overall survival was similar.

At baseline, a higher proportion of patients in the IgRT cohort had experienced SBIs than in the matched no‐IgRT cohort, although exposure to anti‐infectives and immunosuppressants was similar in both cohorts. This may suggest that patients in the IgRT cohort had experienced persistent and/or severe infections that did not improve despite appropriate selection and duration of anti‐infective treatments and/or immunosuppressants (which can leave patients susceptible to infection) before IgRT use. There is also a lack of information on the number of IgRT infusions or the dose received in the IgRT cohort; however, based on the observed data, it may be that these patients received suboptimal and non‐individualized doses of IgRT such that there were no clear improvements in clinical and HCRU outcomes. Relatively small sample sizes in the IgRT and no‐IgRT cohorts after matching may have also limited the opportunity to demonstrate significant differences in outcomes between these cohorts. A common approach in clinical practice is to individualize treatment by measuring serum IgG levels. Thus, after attaining an optimal level, the dose can be adjusted accordingly to achieve serum IgG levels considered to be protective in order to minimize the risk of infections [40–42].

In the current study, 26.9% of patients in the SID cohort experienced at least one infection‐associated hospitalization, with a mean length of hospital stay of 12.9 days. In the same cohort, 92.4% of these patients used anti‐infectives, with antibiotics being the most common type. Furthermore, in those patients with MM and SID, the burden of infection and HCRU were higher in the IgRT cohort than in the no‐IgRT cohort, which potentially indicates that the IgRT cohort is a more vulnerable population. In the current study, in the 12 months after SID diagnosis in the IgRT cohort and no‐IgRT cohort, the mean numbers of infections (any type) were 11.9 and 8.0, respectively, and the percentages of patients with at least one SBI were 52.1% and 31.0%, respectively. These numbers are higher than those reported in a U.S. claims study using the IQVIA PharMetrics Plus database, in which the mean number of bacterial infections for patients with MM after receiving IgRT was 5.7 versus 4.9 for no‐IgRT patients, and the percentage of patients with at least one SBI was 27.5% versus 24.6% for no‐IgRT‐treated patients [43]. Furthermore, in the U.S. claims study, there was no significant difference between the IgRT and no‐IgRT‐treated patients with MM in the adjusted odds ratio of SBIs (0.77; *p* = 0.544) [43].

The mean serum IgG levels at baseline were lower in the no‐IgRT cohort than in the IgRT cohort, and a greater proportion of patients had IgG levels < 5.0 g/L. At baseline, this should have been an indication to treat these patients with IgRT in order to reach the same level of serum IgG as treated patients. IgG thresholds to define hypogammaglobulinemia are somewhat arbitrary and can vary between different laboratories [31, 44]. In this study, the IgG level to define hypogammaglobulinemia was < 5.0 g/L, which may have resulted in more patients with infections being included. This in turn resulted in an increase in anti‐infective use and greater HCRU compared with studies using a lower IgG threshold.

Despite the benefits of IgRT treatment reported in some studies [25–28, 30], clear guidelines on IgRT initiation in MM are lacking [45]. In patients with MM, susceptibility to infection is due to disease‐related immune defects as well as immunosuppression caused by anticancer treatments [4]. In the current study, the complexity, severity, and varying disease course may have contributed to these patients being vulnerable to infections and receiving IgRT after infection had already occurred. Prophylactic IgRT is not routinely recommended in patients with MM [32, 33]; however, it is advised in patients with low IgG receiving CAR‐T cell therapy or bispecific antibody therapy for at least 1 year after treatment or until serum IgG levels are > 400 mg/dL [13, 46]. Additionally, prophylaxis may be considered in patients with severe recurrent bacterial infections and hypogammaglobulinemia [17, 33]. A framework for initiating and managing IgRT in patients with MM starting therapy with T‐cell engagers has recently been proposed [45], although further studies are needed to determine prophylactic use, optimal dosing, monitoring parameters, and routes of administration [17, 45].

Overall survival was lower in patients with SID than those without SID. Despite the outcomes studied being worse typically in the IgRT cohort than in the no‐IgRT cohort, median overall survival in the IgRT group (which may have represented a more vulnerable patient population with SID) was similar to that in patients not receiving IgRT. This is in contrast to a retrospective study in patients with MM in which a significant reduction in infection‐related hospitalizations and improved 5‐year survival was reported among patients who received continuous treatment for a 1‐year period with intravenous IgRT compared with those who did not receive IgRT [47]. Differences in the proportion of patients with severe hypogammaglobulinemia at baseline, MM severity, IgRT dose, and frequency of IgRT administration may account for survival differences between the studies.

A key strength of this study is the use of databases that included both claims and electronic health record data, which is likely to reflect a more robust approach to accurately identifying patients with an SID diagnosis compared with previous studies.

There are several limitations to this study relating to the retrospective design, including potential selection bias and the lack of controls. In order to benefit from the large dataset available from a database with both administrative claims data and health record data, this study design does not allow for detailed and granular analysis of parameters such as IgRT dose that were not captured in the database. There was also potential for miscoding/misclassification owing to the lack of clinical detail within electronic medical records; therefore, data were missing for some variables, disease severity was not well described, with many patients having unspecified disease severity, and data for tumor response status were not available. Information on paraprotein levels was not available: this could lead to immunodeficient patients with IgG MM (with high M‐protein levels) being misclassified into the no‐SID cohort and potentially an overestimation of the infection rates in this cohort. Future analyses could examine patients with hypogammaglobulinemia, stratified by IgG level; however, this level of nuance was not possible in this analysis, owing to the potential for miscoding or misclassification of ICD codes. Data allowing the identification and analysis of opportunistic infections were not available. Guidance for evaluation, identification, and management of SID remains relatively limited.

## 5. Conclusions

Our study indicates that patients with MM who subsequently develop SID have a higher burden of infections, greater consumption of healthcare resources, and lower overall survival than those without SID. Although infection burden was higher for IgRT‐treated patients with SID than no‐IgRT patients, overall survival did not differ. Thus, IgRT may limit the severity of infections in the SID group. IgRT should be considered for infection prevention in patients with SID owing to its potential benefits in improving patient outcomes. However, future research is warranted on the optimal IgRT treatment in this population, including dose, dosing regimen, and infusion parameters. Understanding the burden of illness in SID may help to develop targeted treatments, used earlier in the treatment pathway, potentially alongside first‐line therapies for patients with MM at risk of developing SID. This would thereby improve outcomes for this patient population and in the future may help to define the standards for evaluation, identification, and management of patients with SID.

## Ethics Statement

This retrospective study used de‐identified patient‐level data and was compliant with the Health Insurance Portability and Accountability Act. Therefore, informed consent and institutional review board approval were not required.

## Consent

This retrospective study used de‐identified patient‐level data and was compliant with the Health Insurance Portability and Accountability Act. Therefore, informed consent and institutional review board approval were not required.

## Disclosure

All authors approved the final version of the document.

## Conflicts of Interest

Csaba Siffel, Marta Kamieniak, Kaili Ren, Shirin Ardeshir‐Rouhani‐Fard, and Drishti Shah are employees of Takeda Development Center Americas, Inc., and are Takeda shareholders. Colin Anderson‐Smits was an employee of Takeda Development Center Americas, Inc. at the time of the study. Current affiliation: GileadSciences, Inc., Foster City, California, USA. Joshua Richter has served on speakers’ bureaus for Bristol Myers Squibb and Janssen and has received consulting fees/has served on advisory boards for Adaptive Biotechnologies, AstraZeneca, Bristol Myers Squibb, Celgene, Janssen, Karyopharm Therapeutics, Oncopeptides, Sanofi, Secura Bio, Takeda, and X4 Pharmaceuticals. Matthew S. Davids has received grant support (paid to his institution) and consulting fees from Ascentage Pharma, AstraZeneca, Bristol Myers Squibb, Genentech, MEI Pharma, Pharmacyclics, and TG Therapeutics; grant support (paid to his institution) from Bristol Myers Squibb, Secura Bio, and Surface Oncology; and consulting fees from AbbVie, Adaptive Biotechnologies, Aptitude Health, BeiGene, Bioascend, Celgene, Curio Science, Eli Lilly, Janssen, Merck, Research to Practice, and Takeda.

## Author Contributions

Csaba Siffel, Matthew S. Davids, Colin Anderson‐Smits, Marta Kamieniak, Drishti Shah, and Joshua Richter contributed to the conceptualization of the study; Csaba Siffel, Matthew S. Davids, Colin Anderson‐Smits, Marta Kamieniak, Kaili Ren, Drishti Shah, and Joshua Richter were involved in the design of the work; Csaba Siffel coordinated data acquisition, and Csaba Siffel, Colin Anderson‐Smits, Kaili Ren, Shirin Ardeshir‐Rouhani‐Fard, and Drishti Shah performed statistical analysis of data; all authors contributed toward interpretation of data and were involved in drafting the work and critically reviewing the manuscript.

## Funding

This study was funded by Takeda Development Center Americas, Inc.

## Supporting Information

Additional supporting information can be found online in the Supporting Information section.

## Supporting information


**Supporting Information 1** Supporting Figure 1. Study design.

**Supporting Information 2** Supporting Figure 2. Disposition of patients with ≥ 12‐month follow‐up.

**Supporting Information 3** Supporting Table 1. ICD‐10‐CM diagnosis codes for SBIs.

**Supporting Information 4** Supporting Table 2. Baseline patient demographics and clinical characteristics.

**Supporting Information 5** Supporting Table 3. Baseline patient demographics and clinical characteristics for IgRT and no‐IgRT unmatched and matched cohorts of patients with SID.

## Data Availability

The data that support the findings of this study are available within the Humedica database. These data are not publicly available, but data are available from the corresponding author upon reasonable request and with the permission of Humedica.
