# Supplementary material for: Multiple Myeloma and Secondary Immunodeficiency: A Retrospective Database Analysis Assessing Burden of Infection and Treatment Patterns
Source: Adv Hematol. 2025 Dec 25;2025:5340241. doi: 10.1155/ah/5340241 (PMC12740457; doi:10.1155/ah/5340241)
Supplement: Supplementary file 1 — Supporting Information 1 Supporting Figure 1. Study design. [file AH-2025-5340241-s001.docx]

**SUPPLEMENTARY FIGURE 1** Study design.


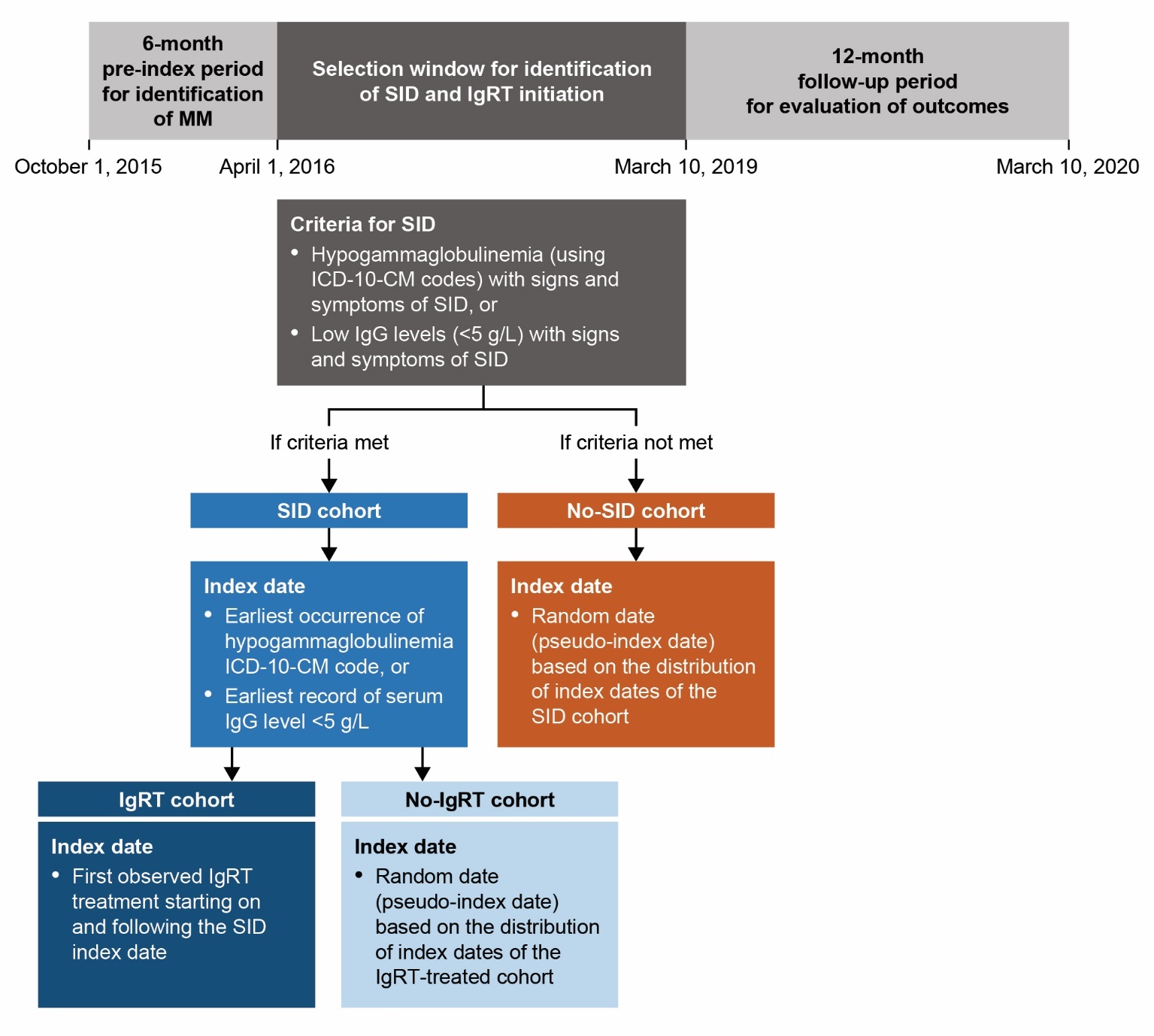


ICD-10-CM, International Classification of Diseases, Tenth Revision, Clinical Modification; IgG, immunoglobulin G; IgRT, immunoglobulin replacement therapy; MM, multiple myeloma; SID, secondary immunodeficiency.

This figure is adapted from a figure previously published by Siffel C, Richter J, Anderson-Smits C, Kamieniak M, Ren K, Shah D, Davids MS. Treatment patterns and burden of infection in patients with chronic lymphocytic leukemia and secondary immunodeficiency: a retrospective database study. *Ann Hematol* 2024;103:4567-4580. doi: 10.1007/s00277-024-05984-6. PMID: 39264434, under the Creative Commons Attribution 4.0 International License, available at http://creativecommons.org/licenses/by/4.0/.
