# Supplementary material for: Multiple Myeloma and Secondary Immunodeficiency: A Retrospective Database Analysis Assessing Burden of Infection and Treatment Patterns
Source: Adv Hematol. 2025 Dec 25;2025:5340241. doi: 10.1155/ah/5340241 (PMC12740457; doi:10.1155/ah/5340241)
Supplement: Supplementary file 2 — Supporting Information 2 Supporting Figure 2. Disposition of patients with ≥ 12‐month follow‐up. [file AH-2025-5340241-s002.docx]

**SUPPLEMENTARY FIGURE 2** Disposition of patients with ≥12-month follow-up.


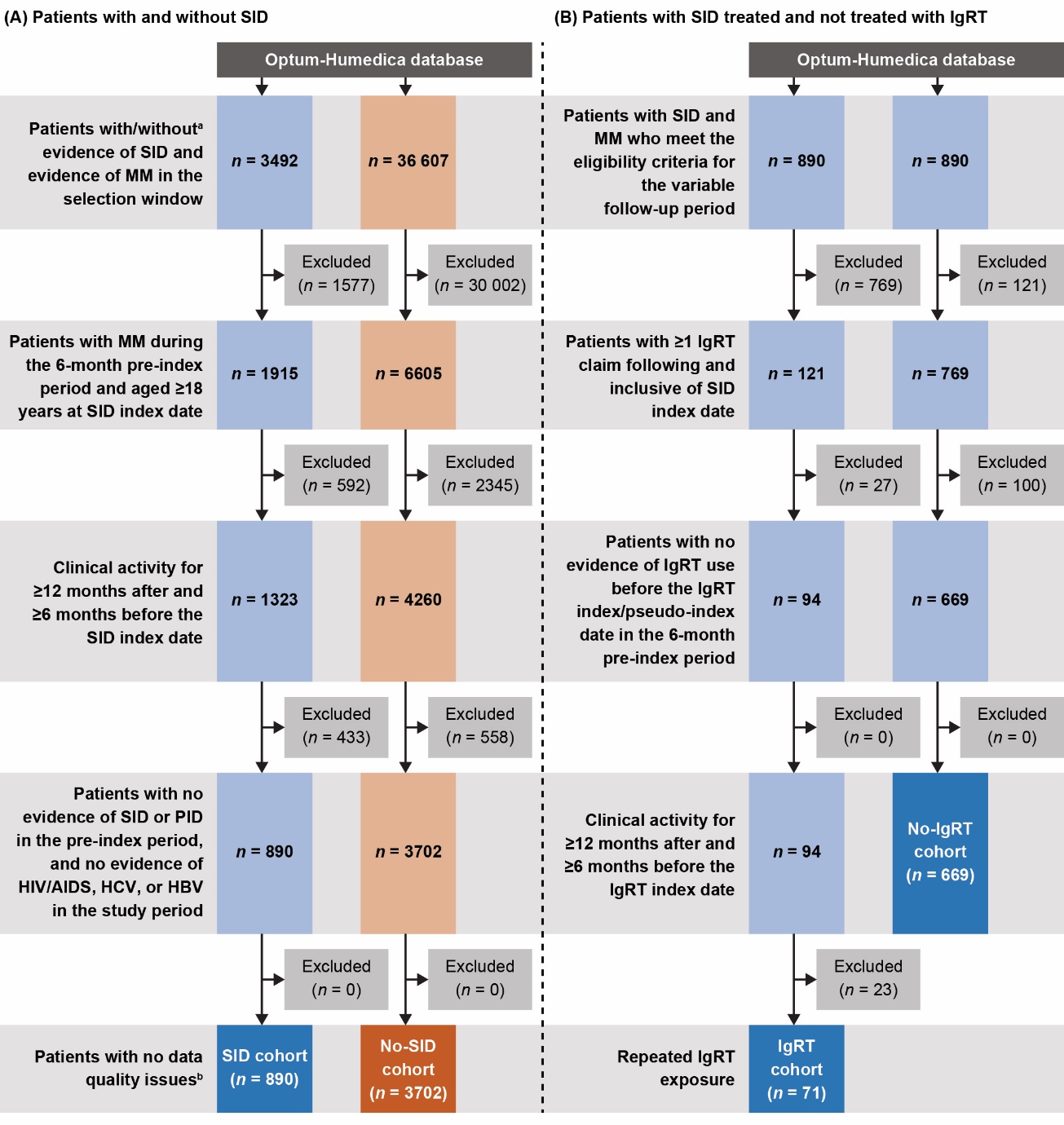


^a^Patients with and without evidence of SID during the selection window (April 1, 2016 to March 10, 2019) were included in the SID cohort (blue boxes) or no-SID cohort (orange boxes), respectively.

^b^Data quality issues were defined as an invalid year of birth or missing sex.

AIDS, acquired immunodeficiency syndrome; HBV, hepatitis B virus; HCV, hepatitis C virus; HIV, human immunodeficiency virus; IgRT, immunoglobulin replacement therapy; MM, multiple myeloma; SID, secondary immunodeficiency.
