# Supplementary material for: Multiple Myeloma and Secondary Immunodeficiency: A Retrospective Database Analysis Assessing Burden of Infection and Treatment Patterns
Source: Adv Hematol. 2025 Dec 25;2025:5340241. doi: 10.1155/ah/5340241 (PMC12740457; doi:10.1155/ah/5340241)
Supplement: Supplementary file 3 — Supporting Information 3 Supporting Table 1. ICD‐10‐CM diagnosis codes for SBIs. [file AH-2025-5340241-s003.docx]

**SUPPLEMENTARY TABLE 1** ICD-10-CM diagnosis codes for SBIs.

| **SBI** | **ICD-10-CM** | **Description** |
| --- | --- | --- |
| Bacteremia or sepsis | A021 | *Salmonella* sepsis |
|  | A227 | Anthrax sepsis |
|  | A267 | *Erysipelothrix* sepsis |
|  | A327 | Listerial sepsis |
|  | A400 | Sepsis due to *Streptococcus*, group a |
|  | A401 | Sepsis due to *Streptococcus*, group b |
|  | A403 | Sepsis due to *Streptococcus pneumoniae* |
|  | A408 | Other streptococcal sepsis |
|  | A409 | Streptococcal sepsis, unspecified |
|  | A4101 | Sepsis due to methicillin-susceptible *Staphylococcus aureus* |
|  | A4102 | Sepsis due to methicillin-resistant *Staphylococcus aureus* |
|  | A411 | Sepsis due to other specified *Staphylococcus* |
|  | A412 | Sepsis due to unspecified *Staphylococcus* |
|  | A413 | Sepsis due to *Haemophilus influenzae* |
|  | A414 | Sepsis due to anaerobes |
|  | A4150 | Gram-negative sepsis, unspecified |
|  | A4151 | Sepsis due to *Escherichia coli* |
|  | A4152 | Sepsis due to *Pseudomonas* |
|  | A4153 | Sepsis due to *Serratia* |
|  | A4159 | Other gram-negative sepsis |
|  | A4181 | Sepsis due to *Enterococcus* |
|  | A4189 | Other specified sepsis |
|  | A419 | Sepsis, unspecified organism |
|  | A427 | Actinomycotic sepsis |
|  | A5486 | Gonococcal sepsis |
|  | B377 | Candidal sepsis |
|  | O0337 | Sepsis after incomplete spontaneous abortion |
|  | O0387 | Sepsis after complete or unspecified spontaneous abortion |
|  | O0487 | Sepsis after (induced) termination of pregnancy |
|  | O0737 | Sepsis after failed attempted termination of pregnancy |
|  | O0882 | Sepsis after ectopic and molar pregnancy |
|  | O85 | Puerperal sepsis |
|  | O8604 | Sepsis after an obstetrical procedure |
|  | R6520 | Severe sepsis without septic shock |
|  | R6521 | Severe sepsis with septic shock |
|  | R7881 | Bacteremia |
|  | T8144XA | Sepsis after a procedure, initial encounter |
|  | T8144XD | Sepsis after a procedure, subsequent encounter |
|  | T8144XS | Sepsis after a procedure, sequela |
| Bacterial meningitis | G000 | *Haemophilus* meningitis |
|  | G001 | Pneumococcal meningitis |
|  | G002 | Streptococcal meningitis |
|  | G003 | Staphylococcal meningitis |
|  | G008 | Other bacterial meningitis |
|  | G009 | Bacterial meningitis, unspecified |
|  | G01 | Meningitis in bacterial diseases classified elsewhere |
| Bacterial pneumonia | J13 | Pneumonia due to *Streptococcus pneumoniae* |
|  | J14 | Pneumonia due to *Haemophilus influenzae* |
|  | J150 | Pneumonia due to *Klebsiella pneumoniae* |
|  | J151 | Pneumonia due to *Pseudomonas* |
|  | J1520 | Pneumonia due to *Staphylococcus*, unspecified |
|  | J15211 | Pneumonia due to methicillin-susceptible *Staphylococcus aureus* |
|  | J15212 | Pneumonia due to methicillin-resistant *Staphylococcus aureus* |
|  | J1529 | Pneumonia due to other *Staphylococcus* |
|  | J153 | Pneumonia due to *Streptococcus*, group b |
|  | J154 | Pneumonia due to other streptococci |
|  | J155 | Pneumonia due to *Escherichia coli* |
|  | J156 | Pneumonia due to other gram-negative bacteria |
|  | J157 | Pneumonia due to *Mycoplasma pneumoniae* |
|  | J158 | Pneumonia due to other specified bacteria |
|  | J159 | Unspecified bacterial pneumonia |
|  | J160 | Chlamydial pneumonia |
|  | J168 | Pneumonia due to other specified infectious organisms |
|  | J17 | Pneumonia in diseases classified elsewhere |
|  | J180 | Bronchopneumonia, unspecified organism |
|  | J181 | Lobar pneumonia, unspecified organism |
|  | J182 | Hypostatic pneumonia, unspecified organism |
|  | J188 | Other pneumonia, unspecified organism |
|  | J189 | Pneumonia, unspecified organism |
| Osteomyelitis | A0224 | *Salmonella* osteomyelitis |
|  | A5443 | Gonococcal osteomyelitis |
|  | B672 | *Echinococcus granulosus* infection of bone |
|  | M4620 | Osteomyelitis of vertebra, site unspecified |
|  | M4621 | Osteomyelitis of vertebra, occipito-atlanto-axial region |
|  | M4622 | Osteomyelitis of vertebra, cervical region |
|  | M4623 | Osteomyelitis of vertebra, cervicothoracic region |
|  | M4624 | Osteomyelitis of vertebra, thoracic region |
|  | M4625 | Osteomyelitis of vertebra, thoracolumbar region |
|  | M4626 | Osteomyelitis of vertebra, lumbar region |
|  | M4627 | Osteomyelitis of vertebra, lumbosacral region |
|  | M4628 | Osteomyelitis of vertebra, sacral, and sacrococcygeal region |
|  | M8600 | Acute hematogenous osteomyelitis, unspecified site |
|  | M86011 | Acute hematogenous osteomyelitis, right shoulder |
|  | M86012 | Acute hematogenous osteomyelitis, left shoulder |
|  | M86019 | Acute hematogenous osteomyelitis, unspecified shoulder |
|  | M86021 | Acute hematogenous osteomyelitis, right humerus |
|  | M86022 | Acute hematogenous osteomyelitis, left humerus |
|  | M86029 | Acute hematogenous osteomyelitis, unspecified humerus |
|  | M86031 | Acute hematogenous osteomyelitis, right radius and ulna |
|  | M86032 | Acute hematogenous osteomyelitis, left radius and ulna |
|  | M86039 | Acute hematogenous osteomyelitis, unspecified radius and ulna |
|  | M86041 | Acute hematogenous osteomyelitis, right hand |
|  | M86042 | Acute hematogenous osteomyelitis, left hand |
|  | M86049 | Acute hematogenous osteomyelitis, unspecified hand |
|  | M86051 | Acute hematogenous osteomyelitis, right femur |
|  | M86052 | Acute hematogenous osteomyelitis, left femur |
|  | M86059 | Acute hematogenous osteomyelitis, unspecified femur |
|  | M86061 | Acute hematogenous osteomyelitis, right tibia and fibula |
|  | M86062 | Acute hematogenous osteomyelitis, left tibia and fibula |
|  | M86069 | Acute hematogenous osteomyelitis, unspecified tibia and fibula |
|  | M86071 | Acute hematogenous osteomyelitis, right ankle and foot |
|  | M86072 | Acute hematogenous osteomyelitis, left ankle and foot |
|  | M86079 | Acute hematogenous osteomyelitis, unspecified ankle and foot |
|  | M8608 | Acute hematogenous osteomyelitis, other sites |
|  | M8609 | Acute hematogenous osteomyelitis, multiple sites |
|  | M8610 | Other acute osteomyelitis, unspecified site |
|  | M86111 | Other acute osteomyelitis, right shoulder |
|  | M86112 | Other acute osteomyelitis, left shoulder |
|  | M86119 | Other acute osteomyelitis, unspecified shoulder |
|  | M86121 | Other acute osteomyelitis, right humerus |
|  | M86122 | Other acute osteomyelitis, left humerus |
|  | M86129 | Other acute osteomyelitis, unspecified humerus |
|  | M86131 | Other acute osteomyelitis, right radius and ulna |
|  | M86132 | Other acute osteomyelitis, left radius and ulna |
|  | M86139 | Other acute osteomyelitis, unspecified radius and ulna |
|  | M86141 | Other acute osteomyelitis, right hand |
|  | M86142 | Other acute osteomyelitis, left hand |
|  | M86149 | Other acute osteomyelitis, unspecified hand |
|  | M86151 | Other acute osteomyelitis, right femur |
|  | M86152 | Other acute osteomyelitis, left femur |
|  | M86159 | Other acute osteomyelitis, unspecified femur |
|  | M86161 | Other acute osteomyelitis, right tibia and fibula |
|  | M86162 | Other acute osteomyelitis, left tibia and fibula |
|  | M86169 | Other acute osteomyelitis, unspecified tibia and fibula |
|  | M86171 | Other acute osteomyelitis, right ankle and foot |
|  | M86172 | Other acute osteomyelitis, left ankle and foot |
|  | M86179 | Other acute osteomyelitis, unspecified ankle and foot |
|  | M8618 | Other acute osteomyelitis, other site |
|  | M8619 | Other acute osteomyelitis, multiple sites |
|  | M8620 | Subacute osteomyelitis, unspecified site |
|  | M86211 | Subacute osteomyelitis, right shoulder |
|  | M86212 | Subacute osteomyelitis, left shoulder |
|  | M86219 | Subacute osteomyelitis, unspecified shoulder |
|  | M86221 | Subacute osteomyelitis, right humerus |
|  | M86222 | Subacute osteomyelitis, left humerus |
|  | M86229 | Subacute osteomyelitis, unspecified humerus |
|  | M86231 | Subacute osteomyelitis, right radius and ulna |
|  | M86232 | Subacute osteomyelitis, left radius and ulna |
|  | M86239 | Subacute osteomyelitis, unspecified radius and ulna |
|  | M86241 | Subacute osteomyelitis, right hand |
|  | M86242 | Subacute osteomyelitis, left hand |
|  | M86249 | Subacute osteomyelitis, unspecified hand |
|  | M86251 | Subacute osteomyelitis, right femur |
|  | M86252 | Subacute osteomyelitis, left femur |
|  | M86259 | Subacute osteomyelitis, unspecified femur |
|  | M86261 | Subacute osteomyelitis, right tibia and fibula |
|  | M86262 | Subacute osteomyelitis, left tibia and fibula |
|  | M86269 | Subacute osteomyelitis, unspecified tibia and fibula |
|  | M86271 | Subacute osteomyelitis, right ankle and foot |
|  | M86272 | Subacute osteomyelitis, left ankle and foot |
|  | M86279 | Subacute osteomyelitis, unspecified ankle and foot |
|  | M8628 | Subacute osteomyelitis, other site |
|  | M8629 | Subacute osteomyelitis, multiple sites |
|  | M8630 | Chronic multifocal osteomyelitis, unspecified site |
|  | M86311 | Chronic multifocal osteomyelitis, right shoulder |
|  | M86312 | Chronic multifocal osteomyelitis, left shoulder |
|  | M86319 | Chronic multifocal osteomyelitis, unspecified shoulder |
|  | M86321 | Chronic multifocal osteomyelitis, right humerus |
|  | M86322 | Chronic multifocal osteomyelitis, left humerus |
|  | M86329 | Chronic multifocal osteomyelitis, unspecified humerus |
|  | M86331 | Chronic multifocal osteomyelitis, right radius and ulna |
|  | M86332 | Chronic multifocal osteomyelitis, left radius and ulna |
|  | M86339 | Chronic multifocal osteomyelitis, unspecified radius and ulna |
|  | M86341 | Chronic multifocal osteomyelitis, right hand |
|  | M86342 | Chronic multifocal osteomyelitis, left hand |
|  | M86349 | Chronic multifocal osteomyelitis, unspecified hand |
|  | M86351 | Chronic multifocal osteomyelitis, right femur |
|  | M86352 | Chronic multifocal osteomyelitis, left femur |
|  | M86359 | Chronic multifocal osteomyelitis, unspecified femur |
|  | M86361 | Chronic multifocal osteomyelitis, right tibia and fibula |
|  | M86362 | Chronic multifocal osteomyelitis, left tibia and fibula |
|  | M86369 | Chronic multifocal osteomyelitis, unspecified tibia and fibula |
|  | M86371 | Chronic multifocal osteomyelitis, right ankle and foot |
|  | M86372 | Chronic multifocal osteomyelitis, left ankle and foot |
|  | M86379 | Chronic multifocal osteomyelitis, unspecified ankle and foot |
|  | M8638 | Chronic multifocal osteomyelitis, other site |
|  | M8639 | Chronic multifocal osteomyelitis, multiple sites |
|  | M8640 | Chronic osteomyelitis with draining sinus, unspecified site |
|  | M86411 | Chronic osteomyelitis with draining sinus, right shoulder |
|  | M86412 | Chronic osteomyelitis with draining sinus, left shoulder |
|  | M86419 | Chronic osteomyelitis with draining sinus, unspecified shoulder |
|  | M86421 | Chronic osteomyelitis with draining sinus, right humerus |
|  | M86422 | Chronic osteomyelitis with draining sinus, left humerus |
|  | M86429 | Chronic osteomyelitis with draining sinus, unspecified humerus |
|  | M86431 | Chronic osteomyelitis with draining sinus, right radius and ulna |
|  | M86432 | Chronic osteomyelitis with draining sinus, left radius and ulna |
|  | M86439 | Chronic osteomyelitis with draining sinus, unspecified radius and ulna |
|  | M86441 | Chronic osteomyelitis with draining sinus, right hand |
|  | M86442 | Chronic osteomyelitis with draining sinus, left hand |
|  | M86449 | Chronic osteomyelitis with draining sinus, unspecified hand |
|  | M86451 | Chronic osteomyelitis with draining sinus, right femur |
|  | M86452 | Chronic osteomyelitis with draining sinus, left femur |
|  | M86459 | Chronic osteomyelitis with draining sinus, unspecified femur |
|  | M86461 | Chronic osteomyelitis with draining sinus, right tibia and fibula |
|  | M86462 | Chronic osteomyelitis with draining sinus, left tibia and fibula |
|  | M86469 | Chronic osteomyelitis with draining sinus, unspecified tibia and fibula |
|  | M86471 | Chronic osteomyelitis with draining sinus, right ankle and foot |
|  | M86472 | Chronic osteomyelitis with draining sinus, left ankle and foot |
|  | M86479 | Chronic osteomyelitis with draining sinus, unspecified ankle and foot |
|  | M8648 | Chronic osteomyelitis with draining sinus, other site |
|  | M8649 | Chronic osteomyelitis with draining sinus, multiple sites |
|  | M8650 | Other chronic hematogenous osteomyelitis, unspecified site |
|  | M86511 | Other chronic hematogenous osteomyelitis, right shoulder |
|  | M86512 | Other chronic hematogenous osteomyelitis, left shoulder |
|  | M86519 | Other chronic hematogenous osteomyelitis, unspecified shoulder |
|  | M86521 | Other chronic hematogenous osteomyelitis, right humerus |
|  | M86522 | Other chronic hematogenous osteomyelitis, left humerus |
|  | M86529 | Other chronic hematogenous osteomyelitis, unspecified humerus |
|  | M86531 | Other chronic hematogenous osteomyelitis, right radius and ulna |
|  | M86532 | Other chronic hematogenous osteomyelitis, left radius and ulna |
|  | M86539 | Other chronic hematogenous osteomyelitis, unspecified radius and ulna |
|  | M86541 | Other chronic hematogenous osteomyelitis, right hand |
|  | M86542 | Other chronic hematogenous osteomyelitis, left hand |
|  | M86549 | Other chronic hematogenous osteomyelitis, unspecified hand |
|  | M86551 | Other chronic hematogenous osteomyelitis, right femur |
|  | M86552 | Other chronic hematogenous osteomyelitis, left femur |
|  | M86559 | Other chronic hematogenous osteomyelitis, unspecified femur |
|  | M86561 | Other chronic hematogenous osteomyelitis, right tibia and fibula |
|  | M86562 | Other chronic hematogenous osteomyelitis, left tibia and fibula |
|  | M86569 | Other chronic hematogenous osteomyelitis, unspecified tibia and fibula |
|  | M86571 | Other chronic hematogenous osteomyelitis, right ankle and foot |
|  | M86572 | Other chronic hematogenous osteomyelitis, left ankle and foot |
|  | M86579 | Other chronic hematogenous osteomyelitis, unspecified ankle and foot |
|  | M8658 | Other chronic hematogenous osteomyelitis, other site |
|  | M8659 | Other chronic hematogenous osteomyelitis, multiple sites |
|  | M8660 | Other chronic osteomyelitis, unspecified site |
|  | M86611 | Other chronic osteomyelitis, right shoulder |
|  | M86612 | Other chronic osteomyelitis, left shoulder |
|  | M86619 | Other chronic osteomyelitis, unspecified shoulder |
|  | M86621 | Other chronic osteomyelitis, right humerus |
|  | M86622 | Other chronic osteomyelitis, left humerus |
|  | M86629 | Other chronic osteomyelitis, unspecified humerus |
|  | M86631 | Other chronic osteomyelitis, right radius and ulna |
|  | M86632 | Other chronic osteomyelitis, left radius and ulna |
|  | M86639 | Other chronic osteomyelitis, unspecified radius and ulna |
|  | M86641 | Other chronic osteomyelitis, right hand |
|  | M86642 | Other chronic osteomyelitis, left hand |
|  | M86649 | Other chronic osteomyelitis, unspecified hand |
|  | M86651 | Other chronic osteomyelitis, right thigh |
|  | M86652 | Other chronic osteomyelitis, left thigh |
|  | M86659 | Other chronic osteomyelitis, unspecified thigh |
|  | M86661 | Other chronic osteomyelitis, right tibia and fibula |
|  | M86662 | Other chronic osteomyelitis, left tibia and fibula |
|  | M86669 | Other chronic osteomyelitis, unspecified tibia and fibula |
|  | M86671 | Other chronic osteomyelitis, right ankle and foot |
|  | M86672 | Other chronic osteomyelitis, left ankle and foot |
|  | M86679 | Other chronic osteomyelitis, unspecified ankle and foot |
|  | M8668 | Other chronic osteomyelitis, other site |
|  | M8669 | Other chronic osteomyelitis, multiple sites |
|  | M868X0 | Other osteomyelitis, multiple sites |
|  | M868X1 | Other osteomyelitis, shoulder |
|  | M868X2 | Other osteomyelitis, upper arm |
|  | M868X3 | Other osteomyelitis, forearm |
|  | M868X4 | Other osteomyelitis, hand |
|  | M868X5 | Other osteomyelitis, thigh |
|  | M868X6 | Other osteomyelitis, lower leg |
|  | M868X7 | Other osteomyelitis, ankle and foot |
|  | M868X8 | Other osteomyelitis, other site |
|  | M868X9 | Other osteomyelitis, unspecified sites |
|  | M869 | Osteomyelitis, unspecified |
| Septic arthritis | M0000 | Staphylococcal arthritis, unspecified joint |
|  | M00011 | Staphylococcal arthritis, right shoulder |
|  | M00012 | Staphylococcal arthritis, left shoulder |
|  | M00019 | Staphylococcal arthritis, unspecified shoulder |
|  | M00021 | Staphylococcal arthritis, right elbow |
|  | M00022 | Staphylococcal arthritis, left elbow |
|  | M00029 | Staphylococcal arthritis, unspecified elbow |
|  | M00031 | Staphylococcal arthritis, right wrist |
|  | M00032 | Staphylococcal arthritis, left wrist |
|  | M00039 | Staphylococcal arthritis, unspecified wrist |
|  | M00041 | Staphylococcal arthritis, right hand |
|  | M00042 | Staphylococcal arthritis, left hand |
|  | M00049 | Staphylococcal arthritis, unspecified hand |
|  | M00051 | Staphylococcal arthritis, right hip |
|  | M00052 | Staphylococcal arthritis, left hip |
|  | M00059 | Staphylococcal arthritis, unspecified hip |
|  | M00061 | Staphylococcal arthritis, right knee |
|  | M00062 | Staphylococcal arthritis, left knee |
|  | M00069 | Staphylococcal arthritis, unspecified knee |
|  | M00071 | Staphylococcal arthritis, right ankle and foot |
|  | M00072 | Staphylococcal arthritis, left ankle and foot |
|  | M00079 | Staphylococcal arthritis, unspecified ankle and foot |
|  | M0008 | Staphylococcal arthritis, vertebrae |
|  | M0009 | Staphylococcal polyarthritis |
|  | M0010 | Pneumococcal arthritis, unspecified joint |
|  | M00111 | Pneumococcal arthritis, right shoulder |
|  | M00112 | Pneumococcal arthritis, left shoulder |
|  | M00119 | Pneumococcal arthritis, unspecified shoulder |
|  | M00121 | Pneumococcal arthritis, right elbow |
|  | M00122 | Pneumococcal arthritis, left elbow |
|  | M00129 | Pneumococcal arthritis, unspecified elbow |
|  | M00131 | Pneumococcal arthritis, right wrist |
|  | M00132 | Pneumococcal arthritis, left wrist |
|  | M00139 | Pneumococcal arthritis, unspecified wrist |
|  | M00141 | Pneumococcal arthritis, right hand |
|  | M00142 | Pneumococcal arthritis, left hand |
|  | M00149 | Pneumococcal arthritis, unspecified hand |
|  | M00151 | Pneumococcal arthritis, right hip |
|  | M00152 | Pneumococcal arthritis, left hip |
|  | M00159 | Pneumococcal arthritis, unspecified hip |
|  | M00161 | Pneumococcal arthritis, right knee |
|  | M00162 | Pneumococcal arthritis, left knee |
|  | M00169 | Pneumococcal arthritis, unspecified knee |
|  | M00171 | Pneumococcal arthritis, right ankle and foot |
|  | M00172 | Pneumococcal arthritis, left ankle and foot |
|  | M00179 | Pneumococcal arthritis, unspecified ankle and foot |
|  | M0018 | Pneumococcal arthritis, vertebrae |
|  | M0019 | Pneumococcal polyarthritis |
|  | M0020 | Other streptococcal arthritis, unspecified joint |
|  | M00211 | Other streptococcal arthritis, right shoulder |
|  | M00212 | Other streptococcal arthritis, left shoulder |
|  | M00219 | Other streptococcal arthritis, unspecified shoulder |
|  | M00221 | Other streptococcal arthritis, right elbow |
|  | M00222 | Other streptococcal arthritis, left elbow |
|  | M00229 | Other streptococcal arthritis, unspecified elbow |
|  | M00231 | Other streptococcal arthritis, right wrist |
|  | M00232 | Other streptococcal arthritis, left wrist |
|  | M00239 | Other streptococcal arthritis, unspecified wrist |
|  | M00241 | Other streptococcal arthritis, right hand |
|  | M00242 | Other streptococcal arthritis, left hand |
|  | M00249 | Other streptococcal arthritis, unspecified hand |
|  | M00251 | Other streptococcal arthritis, right hip |
|  | M00252 | Other streptococcal arthritis, left hip |
|  | M00259 | Other streptococcal arthritis, unspecified hip |
|  | M00261 | Other streptococcal arthritis, right knee |
|  | M00262 | Other streptococcal arthritis, left knee |
|  | M00269 | Other streptococcal arthritis, unspecified knee |
|  | M00271 | Other streptococcal arthritis, right ankle and foot |
|  | M00272 | Other streptococcal arthritis, left ankle and foot |
|  | M00279 | Other streptococcal arthritis, unspecified ankle and foot |
|  | M0028 | Other streptococcal arthritis, vertebrae |
|  | M0029 | Other streptococcal polyarthritis |
|  | M0080 | Arthritis due to other bacteria, unspecified joint |
|  | M00811 | Arthritis due to other bacteria, right shoulder |
|  | M00812 | Arthritis due to other bacteria, left shoulder |
|  | M00819 | Arthritis due to other bacteria, unspecified shoulder |
|  | M00821 | Arthritis due to other bacteria, right elbow |
|  | M00822 | Arthritis due to other bacteria, left elbow |
|  | M00829 | Arthritis due to other bacteria, unspecified elbow |
|  | M00831 | Arthritis due to other bacteria, right wrist |
|  | M00832 | Arthritis due to other bacteria, left wrist |
|  | M00839 | Arthritis due to other bacteria, unspecified wrist |
|  | M00841 | Arthritis due to other bacteria, right hand |
|  | M00842 | Arthritis due to other bacteria, left hand |
|  | M00849 | Arthritis due to other bacteria, unspecified hand |
|  | M00851 | Arthritis due to other bacteria, right hip |
|  | M00852 | Arthritis due to other bacteria, left hip |
|  | M00859 | Arthritis due to other bacteria, unspecified hip |
|  | M00861 | Arthritis due to other bacteria, right knee |
|  | M00862 | Arthritis due to other bacteria, left knee |
|  | M00869 | Arthritis due to other bacteria, unspecified knee |
|  | M00871 | Arthritis due to other bacteria, right ankle and foot |
|  | M00872 | Arthritis due to other bacteria, left ankle and foot |
|  | M00879 | Arthritis due to other bacteria, unspecified ankle and foot |
|  | M0088 | Arthritis due to other bacteria, vertebrae |
|  | M0089 | Polyarthritis due to other bacteria |
|  | M009 | Pyogenic arthritis, unspecified |
| Visceral abscess | D733 | Abscess of spleen |
|  | J851 | Abscess of lung with pneumonia |
|  | J852 | Abscess of lung without pneumonia |
|  | K3521 | Acute appendicitis with generalized peritonitis, with abscess |
|  | K3533 | Acute appendicitis with perforation and localized peritonitis, with abscess |
|  | K50014 | Crohn’s disease of small intestine with abscess |
|  | K50114 | Crohn’s disease of large intestine with abscess |
|  | K50814 | Crohn’s disease of both small and large intestine with abscess |
|  | K50914 | Crohn’s disease, unspecified, with abscess |
|  | K51014 | Ulcerative (chronic) pancolitis with abscess |
|  | K51214 | Ulcerative (chronic) proctitis with abscess |
|  | K51314 | Ulcerative (chronic) rectosigmoiditis with abscess |
|  | K51414 | Inflammatory polyps of colon with abscess |
|  | K51514 | Left-sided colitis with abscess |
|  | K51814 | Other ulcerative colitis with abscess |
|  | K51914 | Ulcerative colitis, unspecified, with abscess |
|  | K5700 | Diverticulitis of small intestine with perforation and abscess without bleeding |
|  | K5701 | Diverticulitis of small intestine with perforation and abscess with bleeding |
|  | K5720 | Diverticulitis of large intestine with perforation and abscess without bleeding |
|  | K5721 | Diverticulitis of large intestine with perforation and abscess with bleeding |
|  | K5740 | Diverticulitis of both small and large intestine with perforation and abscess without bleeding |
|  | K5741 | Diverticulitis of both small and large intestine with perforation and abscess with bleeding |
|  | K5780 | Diverticulitis of intestine, part unspecified, with perforation and abscess without bleeding |
|  | K5781 | Diverticulitis of intestine, part unspecified, with perforation and abscess with bleeding |
|  | K610 | Anal abscess |
|  | K611 | Rectal abscess |
|  | K612 | Anorectal abscess |
|  | K6131 | Horseshoe abscess |
|  | K6139 | Other ischiorectal abscess |
|  | K614 | Intrasphincteric abscess |
|  | K615 | Supralevator abscess |
|  | K750 | Abscess of liver |
|  | N151 | Renal and perinephric abscess |

Abbreviations: ICD-10-CM, International Classification of Diseases, Tenth Revision, Clinical Modification; SBI, severe bacterial infection.
