# Supplementary material for: Multiple Myeloma and Secondary Immunodeficiency: A Retrospective Database Analysis Assessing Burden of Infection and Treatment Patterns
Source: Adv Hematol. 2025 Dec 25;2025:5340241. doi: 10.1155/ah/5340241 (PMC12740457; doi:10.1155/ah/5340241)
Supplement: Supplementary file 4 — Supporting Information 4 Supporting Table 2. Baseline patient demographics and clinical characteristics. [file AH-2025-5340241-s004.docx]

**SUPPLEMENTARY TABLE 2** Baseline patient demographics and clinical characteristics.

|  | SID cohort  (*n* = 890) | No-SID cohort  (*n* = 3702) | *p* value |
| --- | --- | --- | --- |
| **Demographic** |  |  |  |
| Age, years, mean (SD) | 66.5 (10.34) | 68.8 (10.99) | <0.001 |
| Age category, years, *n* (%) |  |  | <0.001 |
| 18–34 | 3 (0.3) | 5 (0.1) |  |
| 35–44 | 16 (1.8) | 67 (1.8) |  |
| 45–54 | 90 (10.1) | 326 (8.8) |  |
| 55–64 | 264 (29.7) | 881 (23.8) |  |
| 65–74 | 309 (34.7) | 1189 (32.1) |  |
| ≥75 | 208 (23.4) | 1234 (33.3) |  |
| Female, *n* (%) | 405 (45.5) | 1772 (47.9) | 0.323 |
| Race, *n* (%) |  |  | <0.001 |
| White/Caucasian | 741 (83.3) | 2700 (72.9) |  |
| Black/African American | 107 (12.0) | 747 (20.2) |  |
| Asian | 3 (0.3) | 42 (1.1) |  |
| Other/unknown | 39 (4.4) | 213 (5.8) |  |
| Ethnicity, *n* (%) |  |  | 0.011 |
| Hispanic/Latino | 35 (3.9) | 129 (3.5) |  |
| Not Hispanic/Latino | 829 (93.1) | 3377 (91.2) |  |
| Unknown | 26 (2.9) | 196 (5.3) |  |
| **Clinical characteristic** |  |  |  |
| Disease severity, *n* (%) |  |  | NR |
| In remission | 58 (6.5) | 444 (12.0) |  |
| In relapse | 69 (7.8) | 139 (3.8) |  |
| Remission not achieved | 763 (85.7) | 3119 (84.3) |  |
| Missing/unknown | NR | NR |  |
| Charlson Comorbidity Index score, mean (SD)^a^ | 4.2 (2.3) | 3.5 (1.9) | <0.001 |
| Duration of cancer, months, mean (SD)^b^ | 13.0 (10.3) | 15.3 (10.1) | <0.001 |
| Infection, *n* (%) |  |  |  |
| Any | 368 (41.3) | 838 (22.6) | <0.001 |
| Severe bacterial infection | 178 (20.0) | 334 (9.0) | <0.001 |
| Exposed to immunosuppressants, *n* (%) | 6 (0.7) | 11 (0.3) | 0.119 |
| Exposed to anti-infectives, *n* (%) | 755 (84.8) | 1752 (47.3) | <0.001 |
| ECOG performance status, *n* (%) |  |  | 0.066 |
| 0 | 118 (13.3) | 320 (8.6) |  |
| 1 | 166 (18.7) | 315 (8.5) |  |
| 2 | 34 (3.8) | 62 (1.7) |  |
| 3 | 4 (0.4) | 14 (0.4) |  |
| 4 | 1 (0.1) | 1 (<0.1) |  |
| Missing | 567 (63.7) | 2990 (80.8) |  |
| Serum IgG level, g/L |  |  |  |
| Mean (SD) | 8.3 (8.12)^c^ | 14.3 (9.96)^d^ | <0.001 |

Baseline characteristics were reported on or as close to the index date as possible. For the SID cohort, the index date was the earliest occurrence of hypogammaglobulimemia or serum IgG level <5.0 g/L. The index date for the no-SOD cohort was a randomly assigned date based on the distribution of index dates in the SID cohort.

^a^Comorbidities of interest were also examined and included congestive heart failure, coronary artery disease, chronic obstructive pulmonary disease, chronic renal disease, diabetes mellitus, hypertension, secondary/other malignancies, thyroid disease, rheumatologic disease, rheumatoid arthritis, cytopenia, idiopathic thrombocytopenia, and renal insufficiency.

^b^Duration of cancer was calculated based on the number of days between the first diagnosis date available in the data set (all available pre-index period) and the index date.

^c^*n* = 634.

^d^*n* = 2003.

Abbreviations: ECOG, Eastern Cooperative Oncology Group; IgG, immunoglobulin G; NR, not reported; SD, standard deviation; SID, secondary immunodeficiency.
