# Supplementary material for: Multiple Myeloma and Secondary Immunodeficiency: A Retrospective Database Analysis Assessing Burden of Infection and Treatment Patterns
Source: Adv Hematol. 2025 Dec 25;2025:5340241. doi: 10.1155/ah/5340241 (PMC12740457; doi:10.1155/ah/5340241)
Supplement: Supplementary file 5 — Supporting Information 5 Supporting Table 3. Baseline patient demographics and clinical characteristics for IgRT and no‐IgRT unmatched and matched cohorts of patients with SID. [file AH-2025-5340241-s005.docx]

**SUPPLEMENTARY TABLE 3** Baseline patient demographics and clinical characteristics for IgRT and no-IgRT unmatched and matched cohorts of patients with SID.

|  | Unmatched cohorts | | | | | Matched cohorts | | | |
| --- | --- | --- | --- | --- | --- | --- | --- | --- | --- |
|  | **IgRT**  **(*n* = 71)** | **No-IgRT**  **(*n* = 669)** | ***p* value^a^** | **SMD** | **IgRT**  **(*n* = 71)** | | **No-IgRT**  **(*n* = 71)** | ***p* value^a^** | **SMD** |
| **Demographic** |  |  |  |  |  | |  |  |  |
| Age, years, mean (SD) | 66.6 (10.3) | 66.5 (10.5) | 0.982 | 0 | 66.6 (10.3) | | 66.0 (10.5) | 0.725 | 0.05 |
| Age category, years, *n* (%) |  |  | 0.773 | 0.14 |  | |  | 0.351 | 0.17 |
| 18–54 | 9 (12.7) | 84 (12.6) |  |  | 9 (12.7) | | 10 (14.1) |  |  |
| 55–64 | 24 (33.8) | 200 (29.9) |  |  | 24 (33.8) | | 21 (29.6) |  |  |
| 65–74 | 20 (28.2) | 227 (33.9) |  |  | 20 (28.2) | | 25 (35.2) |  |  |
| ≥75 | 18 (25.4) | 158 (23.6) |  |  | 18 (25.4) | | 15 (21.1) |  |  |
| Sex, *n* (%) |  |  | 0.9 | −0.02 |  | |  | 0.384 | −0.14 |
| Female | 31 (43.7) | 300 (44.8) |  |  | 31 (43.7) | | 36 (50.7) |  |  |
| Male | 40 (56.3) | 369 (55.2) |  |  | 40 (56.3) | | 35 (49.3) |  |  |
| Race, *n* (%) |  |  | 0.084 | 0.38 |  | |  | 0.687 | 0.35 |
| White/Caucasian | 66 (93.0) | 539 (80.6) |  |  | 66 (93.0) | | 58 (81.7) |  |  |
| Black/African American | 4 (5.6) | 90 (13.5) |  |  | 4 (5.6) | | 9 (12.7) |  |  |
| Asian | 0 | 3 (0.4) |  |  | 0 | | 1 (1.4) |  |  |
| Other/unknown | 1 (1.4) | 37 (5.5) |  |  | 1 (1.4) | | 3 (4.2) |  |  |
| Ethnicity, *n* (%) |  |  | 0.2 | 0.2 |  | |  | 0.801 | 0.17 |
| Hispanic/Latino | 6 (8.5) | 28 (4.2) |  |  | 6 (8.5) | | 3 (4.2) |  |  |
| Not Hispanic/Latino | 64 (90.1) | 617 (92.2) |  |  | 64 (90.1) | | 67 (94.4) |  |  |
| Unknown | 1 (1.4) | 24 (3.6) |  |  | 1 (1.4) | | 1 (1.4) |  |  |
| **Clinical characteristic** |  |  |  |  |  | |  |  |  |
| Disease severity, *n* (%) |  |  | NR | NR |  | |  | NR | NR |
| In remission | 15 (21.1) | 92 (13.8) |  |  | 15 (21.1) | | 6 (8.5) |  |  |
| In relapse | 10 (14.1) | 59 (8.8) |  |  | 10 (14.1) | | 10 (14.1) |  |  |
| Remission not achieved | 46 (64.8) | 505 (75.5) |  |  | 46 (64.8) | | 55 (77.5) |  |  |
| Missing/unknown | 0 | 13 (1.9) |  |  | NR | | NR |  |  |
| Charlson Comorbidity Index score, mean (SD)^b^ | 4.8 (2.43) | 4.2 (2.30) | 0.031 | 0.26 | 4.8 (2.43) | | 4.7 (2.30) | 0.859 | 0.03 |
| Duration of MM, months, mean (SD) | 21.0 (13.1) | 16.0 (10.9) | 0.002 | 0.42 | 21.0 (13.1) | | 20.3 (12.8) | 0.654 | 0.06 |
| Infection, *n* (%) |  |  |  |  |  | |  |  |  |
| Any | 52 (73.2) | 270 (40.4) | <0.001 | 0.7 | 52 (73.2) | | 51 (71.8) | 0.782 | 0.03 |
| Severe bacterial infection | 33 (46.5) | 120 (17.9) | <0.001 | 0.64 | 33 (46.5) | | 21 (29.6) | 0.028 | 0.35 |
| Exposed to immunosuppressants, *n* (%) | 3 (4.2) | 6 (0.9) | 0.047 | 0.21 | 3 (4.2) | | 2 (2.8) | 0.655 | 0.08 |
| Exposed to anti-infectives, *n* (%) | 69 (97.2) | 554 (82.8) | <0.001 | 0.49 | 69 (97.2) | | 68 (95.8) | 0.564 | 0.08 |
| ECOG performance status, *n* (%) |  |  | 0.069 | −0.31 |  | |  | 0.157 | −0.4 |
| 0–2 | 25 (35.2) | 263 (39.3) |  |  | 25 (35.2) | | 28 (39.4) |  |  |
| 3–4 | 2 (2.8) | 3 (0.4) |  |  | 2 (2.8) | | 0 |  |  |
| Missing | 44 (62.0) | 403 (60.2) |  |  | 44 (62.0) | | 43 (60.6) |  |  |
| Serum IgG level, g/L |  |  |  |  |  | |  |  |  |
| Mean (SD) | 5.8 (7.3) | 5.4 (4.3) | 0.64 | 0.07 | 5.8 (7.3) | | 4.97 (3.9) | 0.512 | 0.14 |
| Patients with level <5.0 g/L, *n* (%) | 42 (59.2) | 419 (62.6) | 0.607 | −0.07 | 42 (59.2) | | 55 (77.5) | 0.024 | −0.4 |

^a^Pair-wise comparison of mean or median.

^b^Comorbidities of interest were also examined and included congestive heart failure, coronary artery disease, chronic obstructive pulmonary disease, chronic renal disease, diabetes mellitus, hypertension, secondary/other malignancies, thyroid disease, rheumatologic disease, rheumatoid arthritis, cytopenia, idiopathic thrombocytopenia, and renal insufficiency.

Abbreviations: ECOG, Eastern Cooperative Oncology Group; IgG, immunoglobulin G; IgRT, immunoglobulin replacement therapy; MM, multiple myeloma; NR, not reported; SD, standard deviation; SID, secondary immunodeficiency; SMD, standardized mean difference.
